# The computational psychiatry of reward: broken brains or misguided minds?

**DOI:** 10.3389/fpsyg.2015.01445

**Published:** 2015-09-29

**Authors:** M. Moutoussis, G. W. Story, R. J. Dolan

**Affiliations:** ^1^Wellcome Trust Centre for Neuroimaging, University College LondonLondon, UK; ^2^Centre for Health Policy, Institute of Global Health Innovation, Imperial CollegeLondon, UK; ^3^Max Planck UCL Centre for Computational Psychiatry and Ageing Research, University College LondonLondon, UK

**Keywords:** computational psychiatry, dualism, optimality, psychiatric nosology, Bayesian inference

## Abstract

Research into the biological basis of emotional and motivational disorders is in danger of riding roughshod over a patient-centered psychiatry and falling into the dualist errors of the past, i.e., by treating mind and brain as conceptually distinct. We argue that a psychiatry informed by computational neuroscience, computational psychiatry, can obviate this danger. Through a focus on the reasoning processes by which humans attempt to maximize reward (and minimize punishment), and how such reasoning is expressed neurally, computational psychiatry can render obsolete the polarity between biological and psychosocial conceptions of illness. Here, the term ‘psychological’ comes to refer to information processing performed by biological agents, seen in light of underlying goals. We reflect on the implications of this perspective for a definition of mental disorder, including what is entailed in asserting that a particular disorder is ‘biological’ or ‘psychological’ in origin. We propose that a computational approach assists in understanding the topography of mental disorder, while cautioning that the point at which eccentric reasoning constitutes disorder often remains a matter of cultural judgment.

I’m gonna, I’m gonna lose my baby/So I always keep a bottle near

*[The psychiatrist] said, “I just think you’re depressed.”/This, me, yeah, baby, and the rest*.

A. Winehouse (2007), musician who died of alcohol intoxication in 2011

## Introduction

The idea that reward processing is important in emotional and motivational psychiatric disorders comes from a view of the mind-as-decision-maker. This idea has been developed within the nascent field of computational psychiatry, the clinical offshoot of computational neurobiology. Within this framework, ‘psychiatric disorder’ entails a breakdown in the brain’s inability to optimize decisions. Thus, to the extent that good decisions set up the individual to optimally obtain reward, ‘psychiatric disorder’ entails a suboptimal seeking of reward within an environment. As an approach computational psychiatry promises much by way of future diagnostic and therapeutic applications (Huys et al., 2011; Montague et al., 2012).

We are of course mindful that psychiatry has seen many promising directions that have delivered much less than hoped. In this article we argue that computational psychiatry has already made major contributions in resolving important conceptual divides in mental health. These have been expressed in varying ways but are located around biological/psychological – diagnostic/whole-person polarities (Boyle and Johnstone, 2014; Hayes and Bell, 2014). This has led to a situation where biological research is accused of shocking oversimplification of the mind, and psychosocial research accused of an equally shocking neglect of the brain (‘mindlessness vs. brainlessness’). Intimately related is the question of when psychiatric intervention is justified to address mental symptoms^1^. Here medical professionals may inappropriately diagnose and prosecute biological interventions (Szasz, 1960), while psychological therapists can be just as disempowering (Dolnick, 1998; Romito, 2008). These splits, like old religious conflicts in Europe, concern resource or power struggles among ‘denominations’ as much as they concern disagreements of substance (Bentall, 2009). It is important to note that that a resolution of the latter, to which our present work contributes, may only make slow inroads into the former.

An unhealthy mind is one disposed to make bad decisions and there is no end of examples in psychiatry. Decisions are not just the sine qua non of overt actions, such as a decision of a patient with depression to stay all day in bed, or drink a lethal quantity of vodka and die. We are also ‘deciding’ when we believe a proposition such as ‘my wife has been replaced by a double,’ or believe our senses when they inform that ‘I look fat’ – as in the body image distortion seen in anorexia – right through to a conclusion that ‘the voice is real’ in psychosis. Good decisions on the other hand entail those (among others) that lead to a healthy life, maintain safety and successful reproduction. Computational psychiatry goes further, postulating that *healthy organisms take optimal decisions, given their resources*. ‘Good decisions’ cannot but be those that successfully obtain the ‘best reward,’ those which are good in life and for life. We can call this the Leibnitz^2^ principle – the best possible world of decision-making is with us. Within this framework, ‘psychiatric disorder’ entails an inability to optimize decisions. Thus, to the extent that good decisions set up an individual to optimally obtain reward, ‘psychiatric disorder’ entails a suboptimal reaping of possible reward.

Common sense tells us that motivational and emotional disorders are central to most psychiatric disorders such as drug dependence, clinical depression or schizophrenia. For example, craving is a key motivational disturbance and DSM5 rightly includes it in the 11 criteria of Substance Use Disorder (APA, 2013). In disorders of mood, the symptom of anhedonia is a core criterion of clinical depression, marking it as a motivational and emotional disorder. It is also likely that the fears expressed within persecutory delusions are signs of deeply disordered emotional processing, whereby a diseased brain has recruited basic motivational and emotional mechanisms, originally meant to warn and protect the individual against dire threat, in a completely unwarranted fashion. It may also seem obvious that psychopaths can be construed as individuals inadequately motivated by the pain of others.

At the same time the study of reward in Psychiatry necessitates a widening of the scope of classic computational neurobiology to take seriously the *subjective experience* of motivational and emotional symptoms. Psychiatry is first and foremost a branch of medicine, not of engineering. Psychiatrists recommend biological, psychological and social interventions first and foremost in order to alleviate the suffering of a patient, and those around the patient. Unlike other disciplines, changing people’s behavior is not the final goal but a part – usually a very important part – of restoring health. Conversely, understanding *behavior* motivated by reward and loss is important for psychiatric research. If we were concerned with physical trauma or viral illnesses, a thorough understanding of the body’s mechanisms of immunity and tissue repair would be important, while supporting and correcting such processes would constitute practicing medicine. On the one hand, health research strives to understand both the physiology (the healthy function) and the pathophysiology (function-in-illness) of an underlying biological substrate. On the other, the clinician helps people who suffer as best as possible, while neither over- or under- applying their craft, as condensed in the dictum ‘only the expert surgeon knows when *not* to operate.’ As there is much suffering which medical interventions do not help, much maladaptive behavior and loss-related suffering is within the frame of scientific interest but outside the clinical scope of psychiatry.

Computational psychiatry focuses on those brain-based mechanisms which strive to optimize reward within the environment. We claim that this indivisible coupling of brain-function-environment has already transcended the troublesome polarities of biological vs. psychological, diseased brain vs. maladjusted mind. Furthermore, once an optimizing of function is understood in relation to an individual patient’s needs, the approach also transcends polarities of normative vs. libertarian and reductionist vs. anti-scientific psychiatry. If the study of reward-related decision-making is our new analytic tool, then the goal of this article is to clarify how much emotional and motivational disorders might yield to its explanatory power. Here we also consider foreseeable pitfalls in addition to how this new way of seeing disorder transcends the old polarities that still haunt psychiatry.

## Methods: Review of the Normative Account

One opportunity that a working hypothesis of optimal reward-seeking, given one’s resources, affords is that of normativity. Behavior (be it choice between A and B or free, creative expression) is no longer judged in comparison to a reference sample, a ‘healthy control’ group with all the limitations this entails. Instead behavior is compared to demonstrably optimal solutions in face-valid but solvable tasks.

### The Bayesian Approach

In an *uncertain* world, each piece of *information* is used to *update* the person’s *beliefs* about the *reality underlying appearances* according to this person’s rule-book of *how reality gives rise to appearance*. This is what’s called ‘Bayesian inference’ see **Table 1** for an illustative toy example.

**Table 1:** A toy example of Bayesian reasoning.

Commonsense term	Example	Bayesian term
Belief before considering information (before thinking, not before an event!)	“I am either ‘worthless’ or ‘worthy’ – both are equally likely”	*Prior* belief
Salient information	“My paper was rejected”	Data (or observation)
Rule – how the world works	The worthless get rejected, the worthy are appreciated.	*Generative model* of the world, which provides the *Likelihood* that an underlying state of the world will produce a datum.
Updated belief	“I am worthless”	*Posterior* belief

This toy example does not include decisions about which action to take as yet, only decisions about which state the world is in (here, a self-worth state). Neither motivation nor reward, the central topics of this work are, as yet, explicit.

It is still necessary to write down the stages of information processing leading to normative decisions, and therefore classify where the process may break down in psychiatric disorders. Agents must

(1) have an adequate repertoire of classes (dimensions or categories) that can describe the environment in which they take decisions. ‘Can describe’ here means that beliefs about contexts and states, including prior beliefs, are expressed in terms of this repertoire. Does the set {worthless, worthy} form an adequate repertoire?
(2) have an adequate generative model of what states within the environment can give rise to the observations they make (likelihood of states, including the intentions of other people). Was the rule in the toy example accurate?
(3) be able to invert the generative model so as to determine what the state of the world and the self is likely to be at any given moment. Assuming that the world-view of the toy example was correct, was the update belief warranted or unwarranted?

We now consider decisions about actions, rather than passive beliefs. If, as computational neuroscience claims, brains seek the best possible decisions then they should have values that they seek to optimize, values which are meaningful even if not explicitly represented.

### Utility as Consistent Probability Representation

If the value of different outcomes that can be obtained via different behavioral strategies in a given context is well defined for an agent, we can call these values the ‘utilities’ of the different outcomes and map them to the probability of an agent adopting the corresponding strategy. Rewards are outcomes that reinforce human behavior or are reported as appetitive, desirable, hedonic, pleasant by healthy humans. Confronted with known choices A, B, and C an agent will ascribe ‘utilities’ *u*(A), *u*(B), *u*(C) such that they can choose by applying a well-defined choice probability. For choice A, this would be *π* (A; *u*(A),*u*(B),*u*(C)). Here we operationalize the motivational value of an outcome as the relative (but otherwise consistent) probability with which it is chosen. Let’s call this ‘consistent probability representation’ on the part of the agent. The fact that this can be well-defined is a hypothesis with extremely productive consequences, which we presently describe. It is consistent probability representation that makes it possible to construct a full Bayesian Decision-making psychiatry (BDP), (Montague et al., 2012; Huys et al., 2014). In addition to 1–3 above, agents need -

(4) an adaptive utility function, as just described; and
(5) a generative model that includes an accurate prediction of which outcomes will follow which decision, given a state of the world and the self (model of control), so that they can choose actions that will impact upon the state of the world to produce outcomes with maximum utility.

We immediately note that consistent probability representation firmly maps utility to probability – which is, in Bayesian terms, just another kind of belief (Friston et al., 2013); where the model of control is just part of the generative model. Thus (4) and (5) are not additions to the Bayesian schema but are special cases of its elements.

‘Mental disorder’ can be said to exist when this decision-making apparatus itself is impaired, rather than reflecting any issues with its inputs. Note, however, that the decision-making apparatus by virtue of its Bayesian nature accumulates experience. Every updated belief contains the weight of its priors and forms the prior of the next update. Hence an ‘impairment’ may consist in the development of a decision-making apparatus poorly adapted for the circumstance in question so that there is no firm distinction between maladapted and diseased decision-making apparati. At the same time there is no guarantee that brain development will not encode posteriors into irreversible structure. As an example, the accent with which one speaks is part of the posteriors about the world encoded in childhood. It is very difficult to learn to pronounce a foreign language like a native in adulthood. Hence with respect to an environment where speaking this new language without a foreign accent is optimal, child development has in this broad definition “damaged” the brain.

We can now illustrate this scheme by locating motivational and emotional problems to distinct parts of this apparatus:

(1) Development may not have equipped the patient with an adequate repertoire of classes. A traumatic life which has set the prior probability that others will dislike me as equal to one can be seen to correspond to the extreme of Beck’s notion of a core belief, which says ‘I am worthless.’ In the opening quote, “I’m gonna lose my baby” might be an exemplar of such a (prior) certainty.
(2) The generative model of self and world may be inadequate, leading to wrong estimations of likelihood. For example, one may not have the requisite knowledge that there are common causes of palpitations and shortness of breath other than serious illness, setting the ground for panic anxiety. In the Winehouse quote the dismissal of the psychiatrists’ opinion (‘you’re depressed’) as unlikely to lead to good care may in fact be a sign of such poverty-of-generative-model.
(3) The person may simply be cognitively impaired, so that they can’t work backward from observations to the underlying reality (technically, ‘model inversion’).

As above, focusing at reward contingent on actions yields two further potential problem areas:

(4) They may believe that no decisions are available to them that are associated with dependably good outcomes (e.g., learnt helplessness, OCD).
(5) They may attach too much utility to certain components of a decision (e.g., relief of negative affect by ‘keeping a bottle near’) rather than to others. In the drinking example, if the value of the awful state that will ensue once the drug wears off is discounted, the sufferer heads for a vicious cycle.

However, not all is perfect with utility-maximizing Bayesian schema: human beings appear to violate systematically^3^ the hypothesis of ‘consistent probability representation.’ We now briefly consider an alternative proposal of how people may represent their preferences, which offers a potential solution to these violations.

### Busemeyer: Preference for Reward as a Mixture State

Suppose that decision-making is probed first according to one of three options, for example by asking how much one prefers A over (B or C). We then probe how much B is preferred over C. It turns out that the choice probabilities^4^ (and implied utilities) experimentally measured are not consistent with performing the same experiment in the alternative possible orders. This violation of consistent probability representation – here an order effect – is one of several apparent inconsistencies in probabilistic reasoning that people display. Various explanations has been put forward, ranging from an erroneous bias to invocation of specialized context effects.

But what if before *making* the choice between A, B and C a person does not in fact encode all decision probabilities *π*(A; *u*(A),*u*(B),*u*(C)) etc. ? It may be that their psychological and neurobiological state is better described as the subject being in two or more minds, in a so-called “mixture state” *s* = (*a*(A),*a*(B),*a*(C)) where *a* are the amplitudes of the mixture. In this scenario the process of *making the choice* is implemented as a reduction or projection of the mixture state. The rigorous formalism used to describe the dynamics of mixture states, and what happens at the point of reduction, was first described in quantum physics and recently introduced into decision making by Busemeyer et al. (2009). The reduction or projection process naturally produces order effects: essentially, enquiring about A automatically affects B and C, and so on. The framework is referred to as ‘quantum probability’ (QP) – an unfortunate term in the current context as no physical quanta are involved at all.

The consequences of this framework have not been worked out nearly as fully as, for example, a Bayesian framework. We suggest that the experimental evidence supporting it casts some doubt on the fundamental idea that people represent preference probabilities corresponding to a well defined utility function. There are, however, many cases in which the two frameworks concur (Busemeyer and Bruza, 2012) and so in this instance we proceed with the better-worked-out Bayesian framework, mindful that there are good reasons why its assumptions might provide poor approximations of psychology.

### Marr: Process Models in the Brain

So far we have considered reward and emotion at the level of information processing. Computational psychiatry, however, is not just behavioral economics or behaviorist psychology. Following Marr (1982), we seek evidence that specific, normative, computations we hypothesize are instantiated in neural wetware. This in turn raises the thorny issue that a specific computation – in the sense of a specific normative solution – can be achieved with different problem-solving techniques. It is the signatures of these algorithms that we look for in the neural substrate, and the complete account – from stimulus to neural response, to neural computation to its representation in experimental data – is the ‘process model.’ The best-established process models relevant to the computational psychiatry of reward are arguably those that posit the basal ganglia as representing reward-based learning prediction errors (Seymour et al., 2004) and of the ventral and medial prefrontal cortex representing the values of different actions available to the subject (Rushworth et al., 2011).

### Modeling Motivation and Emotion

There is one issue in motivational and emotional research which has been relatively overlooked within the framework of reward processing, and if unaddressed might reinforce dualist splits. The working definition of motivation within the Bayesian framework appears to claim something trivial, namely defining the motivational power of an outcome as the frequency with which it is chosen. We can choose to call this ‘motivation,’ and this is fine if we were talking about math or physics, where there is no danger of confusing a rigorously defined quantity, say the charge of a quark, with a property of the mind. However, here we are *also* talking about motivation as experienced by patients, so we need to be clear about what sort of claim we are making about the semantic referents to which the term ‘motivation’ belongs. More specifically, are we claiming that the choice-frequency definition of ‘motivation’ is to be taken for granted, while the phenomenal experience of ‘motivation’ is a subject for a future, maybe more optional, clarification or research? This would constitute a linguistic coup d’etat! The hard problem of consciousness need not concern us here: we only need to avoid dualism and – like good Bayesians – optimally combine both linguistic and decision-behavioral evidence.

People place great importance in the distinction between ‘I can’t’ and ‘I don’t care.’ ‘He doesn’t care about me’ is a much more serious accusation than ‘he can’t understand me.’ Yet our measurement of motivation as the currency between observable outcomes and decision probability often makes this distinction quite difficult. Suppose button A gives me a piece of jellied eel four times out of 10, and button B six times out of 10. If I prefer them equally, is it that I am very good as working out frequencies but I don’t care about jellied eel (no motivation), or that I’m very keen on eel but I am incapable of working out frequencies (no ability)? Similarly, if task performance depends on some other psychiatric variable (say on anxiety) we could easily confuse performance at the left side of the Yerkes–Dodson curve (arousal and motivation too low) with performance on the right (high motivation, but arousal detrimentally high). It is not, of course, impossible to distinguish between the ‘I can’t’ and ‘I don’t care’ but ideally both phenomenological and behavioral enquiry are needed. It is interesting to note that the individual’s ‘I can’t’ may be the genetic pool’s ‘I haven’t learnt to appreciate.’

Models traditionally address the issue of motivation-per-outcome by fitting a single parameter (often called ‘temperature’) for each agent. More recently models have parametrized two different aspects of how motivating reward are, even before considering the phenomenological level. The first relates to how often a choice would be made if the reward emanating from it were immediately obtained with great certainty. Even an obviously preferable outcome (‘do you want £5 or £0?’) may not be chosen 100% of the time due, for example, to lapses in attention/misunderstanding. The second aspect has to do with how motivation to make a decision changes as the outcomes of these decisions are, with time, more reliably inferred. This can be seen as an ‘motivational exchange rate’ or ‘decision temperature’ pertaining to a unit change of outcome away from the point of indifference. This pair of concepts is codified as ‘lapse rate and inverse temperature’ in the classic RL temperature (Guitart-Masip et al., 2012) and ‘goal priors and action precision’ in an active inference framework (Friston et al., 2013). Note these are not just different names for the same variables and although they refer to related concepts they have subtly different computational roles.

Although we have a working definition of motivation, we have less of a handle on the term emotion. Our *implied* definition of emotion: a positive or negative utility attaches a value upon the outcomes with which it is associated, and thus upon the states and decisions that lead to them, corresponding to more positive or negative emotional states respectively. Emotion contains *as inseparable parts of each unitary phenomenological state* not only valence and magnitude but rich information about context, intention etc. The desire for sex and the desire for knowledge are not just differently tagged emotions, they are different emotions.

At the moment the way that researchers relate computational variables relate to emotions (if at all) is haphazard; yet tentative progress is being made. In one path breaking study, Rutledge et al. (2014) related changes in subjective well-being to several aspects of a participants’ reward – such as their cumulative reward (‘wealth’), immediate reward and most importantly *immediate reward compared to expectations –* their reward prediction error (RPE). Here changes in subjective wellbeing, ‘how happy do you feel at the moment,’ were best predicted by RPEs. In a bold formulation, Joffily and Coricelli (2013) posited that the phenomenology of several emotions, not just the single dimension of higher vs. lower wellbeing, is intimately linked to *both the temporal dynamics and the certainty* of the beliefs about how one’s state evolves relative to one’s goals or desires. Thus not only does a person feel ‘positive’ as their beliefs shift toward a desired state (as a positive RPE would entail); but this positive emotion has the color of happiness if the current belief is certain but the color of hope if the corresponding belief is uncertain.

This experimental and theoretical progress attests to the feasibility of unifying the ‘client’ (subjective) and the ‘decision-maker’ (objective) perspectives on emotion. The links between the dynamics of reward and the dynamics of emotion show great promise and need a lot of experimental testing, but the first steps of clinical importance have been taken.

## Results

Approaching motivational and emotional disorders through the lens of (computational) reward processing furnishes a number of important results with respect to two of the polarities that have plagued psychiatry but has not made as much progress with respect to a third.

### Biological vs. Psychosocial

Computational psychiatry simultaneously addresses the computational level of what the problem is, the algorithmic level of how it can be operationalized in terms of information processing, and the implementation level in terms of the neural substrate. More practical considerations, such as the behavioral economics of interpersonal exchanges (Camerer, 2003), has obliged scientists to integrate social psychology and neuroscience with basic, or impersonal, reward processing. Let us consider two findings: first, that subjective wellbeing follows RPEs (Rutledge et al., 2014) as above. Second, during interpersonal exchange people may encode both ordinary RPEs (e.g., I’m pleasantly surprised with what she gave me) but also person-representation prediction errors [She will be pleasantly surprised about me, as I’m about to reciprocate generously (Xiang et al., 2012)]. If ordinary RPEs drive some aspects of emotion, it would be strange indeed if person-representation RPEs were unrelated to the strong emotions we experience in an interpersonal sphere: for example, their fragility in emotionally unstable personality or their presumed dearth in psychopathy.

On the other hand, of course, we are far from elucidating the actual way in which social emotions and non-social emotions are represented in their neurological substrates and, inversely, how social and non-social emotional processing changes this substrate, be it through trauma (Chen and Etkin, 2013), learning in psychosis (Murray et al., 2008) or subtle plasticity (Garvert et al., 2015). Signatures of biased reward processing have been found in several disorders but they are far from explaining these disorders either in the sense of explaining symptoms in the here and now or in the sense of predicting the course of the disorder much better than traditional methods (Whelan et al., 2014).

### Disease vs. Maladjustment

Learning about reward takes place at different levels of information processing. Let us consider the example of psychosis. The early, and celebrated, aberrant salience hypothesis of psychotic disorders (Kapur et al., 2005) postulated a disease level wherein dopamine discharges might be epileptic-like, unrelated to information processing, leading to the establishment of psychotic associations (both beliefs and choices) at the phenomenological and behavioral levels. Such an account separates the diseased brain reporting aberrant increased salience; and the healthy brain downstream that tries to make sense of this abnormal salience. However no epileptiform activity has been demonstrated. Increased aberrant salience has been demonstrated in association with schizotypy in healthy individuals and in medicated patients with delusions (Roiser et al., 2009); however, it does not seem to be prominent in prepsychotic and early psychotic states, where no changes in aberrant salience have been found so far (Smieskova et al., 2015). At the same time there is evidence that exaggerated dopamine reactivity to stress is associated with psychotic experiences in predisposed individuals (Hernaus et al., 2015).

Therefore the evidence points toward disease being an overall brain-state, the result of adjustment to psychobiological challenges performed by the individual’s neural phenotype. Computationally, this is inference about salient stimuli at the developmental timescale; while genetically it is likely to be based on ‘intermediate phenotypes,’ e.g., of atypical connectivity (Cao et al., 2016). We can see that this framework renders the dualistic view of ‘disease’ and ‘maladjustment’ obsolete. The canonical teaching of an illness being explained in terms of predisposing, precipitating and perpetuating factors fits much more comfortably with the dynamical view of computational psychiatry, wherein dopamine reactivity or the interplay of prior and posterior beliefs are meaningful (if suboptimal) at different but intimately linked Marrian levels. The computational models of Ruppin and coworkers (Horn and Ruppin, 1994) illustrate a beautiful early example of such thinking. They suggested that the brain performed compensatory adjustments to long-range dysconnectivity in order to preserve the ability to activate appropriate perceptions in response to stimuli. However, these compensatory adjustments result in a propensity for percepts that bear small correlation to stimuli (i.e., hallucinations) to arise. Neurobiological and computational research has greatly refined these insights. We close this brief foray into psychosis research by point out a promising theme relevant to the role of reward, the focus of this issue. From the early theories of dopamine-dependent signal-to-noise (Cohen and Servan-Schreiber, 1992; Servan-Schreiber et al., 1996) to the influential analysis of the role of precision at sensory vs. cognitive levels (Adams et al., 2013) to the findings of exaggerated dopamine reactivity to stress (Hernaus et al., 2015), psychosis has been about aberrations and compensatory changes in synaptic gain. The original aberrant salience theory of psychosis has opened new horizons regarding the role of reward- and threat- anticipation in psychosis; yet it may be the increasingly sophisticated understanding of synaptic gain, especially in its guise as precision calculated in cortical NMDA fields (Adams et al., 2013), that helps us go beyond the oversimplified aspects of salience theory.

At a theoretical level some biological factors are so dominant that to call them ‘predisposing factors’ is misleading (e.g., Down’s syndrome causing Alzheimer’s disease). These can be thought of as maladjustments at another level of the hierarchy – where an evolving reproductive apparatus has not learnt to avoid trisomies. Such maladjustments may be chance events or indeed the result of optimizing compromises between priorities.

At the same time the normative view of reward processing contains an ambiguity that needs acknowledgment and resolution. This is that for *any* input-output behavioral pattern a cost structure can be found for which this pattern of behavior is optimal (Daunizeau et al., 2010). For *any* behavior we can simply say that the person in question emits it because it genuinely optimizes their happiness. This is analogous to the psychological assertion that a patient ‘refuses to change because it would be too painful for them,’ or that an addict or pedophile simply finds indulging too rewarding to trade it against an alternative. Given a conception of what is valuable, e.g., making the most money, we can offer to explain how people attempt to optimize their behavior, and which parts of the process may go wrong. In current practice most research that investigates abnormalities of reward processing takes as a starting point an assumption that there are rewards out there, which have a normative relationship with the individual’s behavior and that peop*le should* value a*nd should* seek. When a rat or human are hungry, two lumps of sugar are more rewarding than one, and we can measure how much harder subjects are willing to work for the chance to get them. We have a normative yardstick: our subject should work just hard enough to maximize the utility of (sugar + effort). Motivational disorder is then defined as a statistically significant deviation from this norm. However, in the real world it is hard to know what people *should* care about and computational, biological and psychosocial research agendas could do well to take seriously what we don’t know.

Computational psychiatry does not do as well, as yet, when dealing with the complexity of human emotion. The problem is acute not because we should address emotion in its huge complexity, but because we have so far dealt with it by a simplification into positive vs. negative emotion, albeit tagged according to experimental tasks in question. If human emotion relevant to psychopathology contains multiple facets as inseparable parts of a phenomenological states, and if these rich states have computational relevance, then current studies are likely to be very remote from actual clinical relevance. Paradiso and Rudrauf (2012) put it eloquently: “… the fear experienced by a mountain climber in potential danger has levels of social complexity unlikely to be reached in mice. In addition to fearing his own end, the mountain climber anticipating a possible death is equally likely also to be scared of losing his spouse and children, leaving them fatherless and exposed to dangers, of the financial consequences of his death on them, of the emotional effects on his parents, and so on. He may simultaneously experience shame (another social emotion) and danger (perhaps toward his self) for having neglected what he thinks were routine safety measures. A human facing the possibility of ceasing to exist has emotions that encompass the inescapable social nature and interconnectedness of our species and multiple levels of self-representation and projection.” We don’t really know which complex emotional constellations found in psychiatric disorders are most relevant, especially for decision-making that can be considered pathological. At the moment we haven’t developed a good way of addressing this most important question scientifically either.

## Discussion

A computational psychiatry of emotional disorders has begun to put on the table key issues that have plagued psychiatry. It provides a framework for bridging biological-psychological-social divides and offers novel perspectives on the question of emotional-motivational ‘diseases’ versus ‘problems.’ This is rendered possible by formulating disorders of motivation and emotion within a normative probabilistic framework which offers sophisticated and neurobiologically plausible accounts of how reward motivate decisions. Many challenges remain. Phenomenology is only tentatively connected to computation; much-promising theoretical concepts have not been put to experimental test, while their normative basis is not understood. For example, we have no rigorous normative account of what utility structures correspond to mental health. A key example is how reward *should* be discounted in the face of time (inter-temporal discounting), valence (complex discounting of negative future events, including dread) or social distance (social discounting). Therefore the statistical connections that have been found between temporal discounting and addictive disorders lack a true normative basis.

Let us now consider a libertarian (or Szaszian) critique of reward processing as a basis for psychiatric research. Szasz protested against a medicalization of deviant behavior, believing that so called psychiatric disorders lack an adequate biological basis. Hence ‘medicalizing’ unjustifiably transgressed peoples’ autonomy (Szasz, 1960). Deciding *a priori* what reward people should value more (as manifested in their choices) or what reward they should care about (as manifested in their phenomenology) is just as much ‘playing god’ once we move beyond trivial choices: in many cases psychiatrically relevant situations are complex enough to negate a dream of finding a normative standard against which to measure motivational disorder. Reward processing should maximize long-term outcomes and so in research practice we use paradigms that have well-defined ends or may be thought of as going on ‘for ever’ (as for example near the beginning of a task with hundreds of trials). Yet what sorts of long-term outcomes are involved in the long-term reward processing important for psychiatry? Individual reproductive fitness? We have no clear idea, and the temptation is to import convenient social norms, rendering our framework only pseudo-normative. Even in the simple example of working for lumps of sugar, mentioned above, there will usually be *some* evaluation of effort and sugar^5^ that renders behavior optimal. This evaluation may be normative with respect to the person’s history, not the task. In any psychiatrically relevant situation considerations rapidly multiply. For example, what if our hungry human is overweight? And what if the reproductive fitness associated with slimness (attracting mates) is socially constructed?

Thomas Szasz and the libertarian tradition (to which the authors belong) argue that rather than impose norms on people – say about which reward would maximize their life expectancy, their reproductive success – we should respect the priorities they have and, by definition, accept a person’s autonomy to seek their own reward by deploying their own motivational structures. So is there no such thing as a motivational or emotional disorder and in fact everyone is just doing the best they can? Szasz would claim that the dream of aberrant reward processing pinning down what’s essential about motivational and emotional disorders is no more solid than the ‘chemical imbalance’ theory of depression or of Freud’s ‘unconscious motivation’ theory of mental illness. To be more specific if the Reward processing domain of the otherwise promising ‘Research Domain Criteria’ framework (Casey et al., 2013) is applied too simplistically we may end up with exactly the same mistakes as in previous biological or psychoanalytic normative straightjackets.

If a Szaszian position simply accepts peoples’ choices for what they are, its extreme opposite would be a 1984 world where people have been taught through social, psychological and biological interventions, not only *what to decide* but actually *what to desire.* While we recoil from the Szaszian extreme as it is dismissive of the importance of psychiatric suffering, psychiatrists cannot dictate what patients should care about – even about their symptoms. The so-called recovery movement can already teach computational neuroscientists that the rewards that patients really care about are not so much to do with their symptoms as with their life goals and values. In that case perhaps the priorities for researching archetypal motivational disorders like depression are not about ‘what motivational disturbance underpins depression’ but ‘what decision structures of the depressed can help them fulfill their values’ (Hayes et al., 1999). Here we have dialectic, because the scientific baby should not be thrown away with the essentialist bath water. The clinician could bring to the patient a biopsychosocial assessment of ‘wrong priors,’ ‘wrong models,’ or ‘wrong utilities.’ They would then decide *in dialog with a patient*, with a diagnosis of say the successor of ‘Depressive Episode,’ now defined in computational terms, what key needs must be targeted and optimized. When it comes to the severe mental illnesses, formulations that go beyond ‘Schizophrenia,’ or indeed ‘Abnormal Salience syndrome’ (Van Os, 2009) will help clinicians and patients consider emotional and motivational dispositions both as threats and as instruments toward recovery. Of course this account assumes patients with some capacity to consider the issues in question, which may itself be severely compromised – for example in acute psychosis.

Why bring in the concept of need when considering reward and emotional disorder? Because biologically reward is not an end in itself, but a good surrogate toward longer-term biological goals. The stability properties of a self-perpetuating system, like a species in an ecosystem, can be conceptualized in terms of having the ‘purpose’ or ‘goal’ to keep perpetuating the system (e.g., the species). One has to be careful philosophically to avoid false teleological justifications, but in the first instance this is small print. We assert that there are physiological homeostatic needs, reproductive/sexual needs, and more complex ones such as needs for social contact. Furthermore, people are motivated by reward that extend beyond their own lives. They will often, in fact, sacrifice their life for much less than ‘two brothers or four cousins,’ as mathematical evolutionary biologists have put it (Maynard Smith, 1993). Each of these needs entails goals, desires and reward; all are relevant to psychiatry; but probably few can be the target of fruitful intervention for each particular patient.

## Conclusion: Computational Psychiatry must be Profoundly Biopsychosocial

In the best possible world scientists will take seriously the question of what needs really matter for patients, what reward form the best surrogates or milestones toward the fulfillment of such needs and will do so in open collaboration with relevant stakeholders. At first sight the rigorous, biologically based discipline of computational psychiatry seems far from patients’ expressed needs, yet the fact that it puts reward and motivation at the center of understanding psychiatric disorder gives it a privileged vantage point toward serving patients.

## Conflict of Interest Statement

The authors declare that the research was conducted in the absence of any commercial or financial relationships that could be construed as a potential conflict of interest.

## Footnotes

1 A most curious term: a symptom without a mental dimension is not a symptom but a sign.

2 Leibnitz claimed that we live in the best world that could possibly (logically, self-consistently) exist. He was famously satirized by Votaire in ‘Candide’ [en.wikipedia.org/wiki/Candide], exemplifying the normativity-pathology dialectic that is highly relevant to us.

3 Though not grossly.

4 Consistent choice utilities map to consistent choice probabilities. In a Bayesian world we would require such consistency of utility. In reality the decision-making processes people use mean that this isn’t so. This is called ‘violation of revealed preference theory.’

5 i.e., the person’s goal or preference priors: Friston et al. (2013).

## References

[B1] Adams R. A. Stephan K. E. Brown H. R. Frith C. D. Friston K. J. (2013). The computational anatomy of psychosis. *Front. Psychiatry* 4:47 10.3389/fpsyt.2013.00047 PMC3667557 23750138

[B2] APA (2013). *DSM-5 Diagnostic and Statistical Manual of Mental Disorders.* Arlington, TX: American Psychiatric Association.

[B3] Bentall R. (2009). *Doctoring the Mind: Is Our Current Treatment of Mental Illness Really any Good?* New York, NY: NYU Press.

[B4] Boyle M. Johnstone L. (2014). Alternatives to psychiatric diagnosis. *Lancet Psychiatry* 1 409–411. 10.1016/S2215-0366(14)70359-1 26361183

[B5] Busemeyer J. Bruza P. (2012). *Quantum Models of Cognition and Decision.* Cambridge: Cambridge University Press 10.1017/CBO9780511997716

[B6] Busemeyer J. Wang Z. Lambert-Mogiliansky A. (2009). Empirical comparison of Markov and quantum models of decision making. *J. Math. Psychol.* 53 423–433. 10.1016/j.jmp.2009.03.002

[B7] Camerer C. F. (2003). *Behavioral Game Theory: Experiments in Strategic Interaction.* Princeton, NJ: Princeton University Press.

[B8] Cao H. Dixson L. Meyer-Lindenberg A. Tost H. (2016). Functional connectivity measures as schizophrenia intermediate phenotypes: advances, limitations, and future directions. *Curr. Opin. Neurobiol.* 36 7–14. 26276700 10.1016/j.conb.2015.07.008

[B9] Casey B. J. Craddock N. Cuthbert B. N. Hyman S. E. Lee F. S. Ressler K. (2013). DSM-5 and RDoC: progress in psychiatry research? *Nat. Rev.* 4 810–814. 10.1038/nrn3621 PMC4372467 24135697

[B10] Chen A. Etkin A. (2013). Hippocampal network connectivity and activation differentiates post-traumatic stress disorder from generalized anxiety disorder. *Neuropsychopharmacology* 38 1889–1898. 10.1038/npp.2013.122 23673864 PMC3746693

[B11] Cohen J. D. Servan-Schreiber D. (1992). Context, cortex, and dopamine: a connectionist approach to behavior and biology in schizophrenia. *Psychol. Rev.* 99 45–77. 10.1037/0033-295X.99.1.45 1546118

[B12] Daunizeau J. den Ouden H. E. M. Pessiglione M. Kiebel S. J. Friston K. J. Stephan K. E. (2010). Observing the observer (II): deciding when to decide. *PLoS ONE* 5:e15555 10.1371/journal.pone.0015555 PMC3001882 21179484

[B13] Dolnick E. (1998). *Madness on the Couch: Blaming the Victim in the Heyday of Psychoanalysis.* New York, NY: Simon and Schuster.

[B14] Friston K. J. Schwartenbeck P. FitzGerald T. Moutoussis M. Behrens T. Dolan R. J. (2013). The anatomy of choice: active inference and agency. *Front. Hum. Neurosci.* 7:598 10.3389/fnhum.2013.00598 PMC3782702 24093015

[B15] Garvert M. M. Moutoussis M. Kurth-Nelson Z. Behrens T. Dolan R. J. (2015). Learning-induced plasticity in medial prefrontal cortex predicts preference malleability. *Neuron* 85 418–428. 10.1016/j.neuron.2014.12.033 25611512 PMC4306543

[B16] Guitart-Masip M. Huys Q. Fuentemilla L. Dayan P. Duzel E. Dolan R. (2012). Go and no-go learning in reward and punishment: interactions between affect and effect. *NeuroImage* 62 154–166. 10.1016/j.neuroimage.2012.04.024 22548809 PMC3387384

[B17] Hayes J. Bell V. (2014). Diagnosis: one useful method among many. *Lancet Psychiatry* 1 412–413. 10.1016/S2215-0366(14)70399-2 26361184

[B18] Hayes S. C. Strosahl K. D. Wilson K. G. (1999). *Acceptance and Commitment Therapy: An Experiential Approach to Behavior Change.* New York, NY: Guildford.

[B19] Hernaus D. Collip D. Lataster J. Viechtbauer W. Myin E. Ceccarini J. (2015). Psychotic reactivity to daily life stress and the dopamine system: a study combining experience sampling and [^18^F]fallypride positron emission tomography. *J. Abnorm. Psychol.* 124 27–37. 10.1037/abn0000010 25688430

[B20] Horn D. Ruppin E. (1994). Synaptic compensation in attractor neural networks: modeling neuropathological findings in schizophrenia. *Neural Computation* (in press).

[B21] Huys Q. Guitart-Masip M. Dayan P. (2014). Decision theoretic psychiatry. *Clin. Psychol. Sci.* 3 374–377.

[B22] Huys Q. Moutoussis M. Willams J. (2011). Are computational models of any use to psychiatry? *Neural Netw.* 24 544–551. 10.1016/j.neunet.2011.03.001 21459554

[B23] Joffily M. Coricelli G. (2013). Emotional valence and the free-energy principle. *PLoS Comput. Biol.* 9:e1003094 10.1371/journal.pcbi.1003094 PMC3681730 23785269

[B24] Kapur S. Mizrahi R. Li M. (2005). From dopamine to salience to psychosis–linking biology, pharmacology and phenomenology of psychosis. *Schizophr. Res.* 79 59–68. 10.1016/j.schres.2005.01.003 16005191

[B25] Marr D. (1982). *Vision.* New York, NY: Freeman.

[B26] Maynard Smith J. (1993). *The Theory of Evolution.* Cambridge: Cambridge University Press.

[B27] Montague P. R. Dolan R. J. Friston K. J. Dayan P. (2012). Computational psychiatry. *TICS* 16 72–80. 10.1016/j.tics.2011.11.018 PMC3556822 22177032

[B28] Murray G. Corlett P. Clark L. Pessiglione M. Blackwell A. Honey G. (2008). Substantia nigra/ventral tegmental reward prediction error disruption in psychosis. *Mol. Psychiatry* 13 267–276. 10.1038/sj.mp.4002058 PMC2564111 17684497

[B29] Paradiso S. Rudrauf D. (2012). Struggle for life, struggle for love and recognition: the neglected self in social cognitive neuroscience. *Dialogues Clin. Neurosci.* 14 65–75. 22577306 10.31887/DCNS.2012.14.1/sparadiso PMC3341651

[B30] Roiser J. P. Stephan K. E. den Ouden H. E. M. Barnes T. R. E. Friston K. J. Joyce E. M. (2009). Do patients with schizophrenia exhibit aberrant salience? *Psychol. Med.* 39 199–209. 10.1017/S0033291708003863 18588739 PMC2635536

[B31] Romito P. (2008). *A Deafening Silence: Hidden Violence Against Women and Children.* Bristol: Policy Press.

[B32] Rushworth M. Noonan M. Boorman E. Walton M. Behrens T. (2011). Frontal cortex and reward-guided learning and decision-making. *Neuron* 70 1054–1069. 10.1016/j.neuron.2011.05.014 21689594

[B33] Rutledge R. Skandali N. Dayan P. Dolan R. (2014). A computational and neural model of momentary subjective well-being. *Proc. Natl. Acad. Sci. U.S.A*. 111 12252–12257. 10.1073/pnas.1407535111 25092308 PMC4143018

[B34] Servan-Schreiber D. Cohen J. D. Steingard S. (1996). Schizophrenic deficits in the processing of context. A test of a theoretical model. *Arch. Gen. Psychiatry* 53 1105–1112. 10.1001/archpsyc.1996.01830120037008 8956676

[B35] Seymour B. O. Doherty J. P. Dayan P. Koltzenburg M. Jones A. K. Dolan R. J. (2004). Temporal difference models describe higher-order learning in humans. *Nature* 429 664–667. 10.1038/nature02581 15190354

[B36] Smieskova R. Roiser J. P. Chaddock C. A. Schmidt A. Harrisberger F. Bendfeldt K. (2015). Modulation of motivational salience processing during the early stages of psychosis. *Schizophr. Res.* 166 17–23. 10.1016/j.schres.2015.04.036 25999039

[B37] Szasz T. (1960). The myth of mental illness. *Am. Psychol.* 15 113–118. 10.1037/h0046535

[B38] Van Os J. (2009). ‘Salience syndrome’ replaces ‘schizophrenia’ in DSM-V and ICD-11: psychiatry’s evidence-based entry into the 21st century? *Acta Psychiatrica Scandinavica* 120 363–372. 10.1111/j.1600-0447.2009.01456.x 19807717

[B39] Whelan R. Watts R. Orr C. Althoff R. Artiges E. Banaschewski T. (2014). Neuropsychosocial profiles of current and future adolescent alcohol misusers. *Nature* 512 185–189. 10.1038/nature13402 25043041 PMC4486207

[B40] Xiang T. Ray D. Lohrenz T. Dayan P. Montague P. R. (2012). Computational phenotyping of two-person interactions reveals differential neural response to depth-of-thought. *PLoS Comput. Biol.* 8:e1002841 10.1371/journal.pcbi.1002841 PMC3531325 23300423

